# Efficacy and Safety of Octreotide for Gastrointestinal Bleeding Due to Portal Hypertension in Children—A Systematic Review

**DOI:** 10.3390/ph19070978

**Published:** 2026-06-24

**Authors:** Ann Kozak, Grace Nolder, Giusy Ranucci, Alessio Provenzani

**Affiliations:** 1 Department of Pharmacy and Therapeutics, University of Pittsburgh School of Pharmacy, Pittsburgh, PA 15261, USA; ack74@pitt.edu (A.K.); gmn22@pitt.edu (G.N.); 2 IRCCS ISMETT, 90127 Palermo, Italy; granucci@ismett.edu; 3 UPMC Italy, 90133 Palermo, Italy

**Keywords:** octreotide, pediatric patients, portal hypertension, gastrointestinal hemorrhage, efficacy, adverse drug reaction, safety, tolerability, drug safety

## Abstract

**Background**: Portal hypertension can lead to complications such as ascites, hepatic encephalopathy, esophageal varices, and gastrointestinal (GI) bleeding, all of which are associated with significant morbidity and mortality. Variceal bleeding is the most severe complication, with an estimated mortality of up to 30%. In children, evidence-based guidelines for the management of GI bleeding secondary to portal hypertension are lacking. In this con-text, octreotide, a synthetic somatostatin analog approved for other indications, has been increasingly used off-label and represents a paradigmatic example of drug re-purposing in pediatrics. **Methods**: Following the 2020 PRISMA guidelines, this systematic review evaluated the efficacy and safety of octreotide for the treatment of portal hyperten-sion-related GI bleeding in children. A comprehensive search of six sources, including five bibliographic databases (PubMed, Embase, Web of Science, Cochrane Library, and EBSCOhost) and the ClinicalTrials.gov registry, was conducted to identify studies in-cluding pediatric patients with GI bleeding secondary to portal hypertension. **Results**: Three non-randomized observational studies were included, assessing bleeding recurrence, packed red blood cell requirements, and adverse events following octreotide admin-istration. Overall, 33 patients were analyzed, with a mean age of 6.3 years. One study reported a reduction in rebleeding episodes and transfusion requirements after oc-treotide treatment. Across all included studies, no serious adverse events were ob-served; mild and reversible hyperglycemia was the only reported drug-related effect. Quantitative synthesis was not feasible due to substantial heterogeneity, missing data, and a serious risk of bias, resulting in very low certainty of evidence. **Conclusions**: Octreotide may represent a feasible therapeutic option for portal hypertension-related GI bleeding in children; however, further prospective and standardized studies are needed to establish its long-term safety and efficacy.

## 1. Introduction

Portal hypertension, characterized by increased pressure in the portal circulation, can lead to several complications, including ascites, hepatic encephalopathy, esophageal varices, and gastrointestinal (GI) bleeding, all of which carry a significant burden of morbidity and mortality [1,2]. The most common etiology of portal hypertension is cirrhosis, accounting for upwards of 90% of cases [3]. Portal hypertension can also be caused by several other conditions, including, but not limited to, portal vein thrombosis, acute hepatitis, liver metastasis, congenital hepatic fibrosis, and congestive heart failure [1,3]. Specifically, in the pediatric population, leading causes of portal hypertension include liver diseases like biliary atresia, infection, intra-abdominal abscess, and portal vein obstruction [4]. It is estimated that liver disease is attributable to two million deaths per year and 4% of all deaths globally [5].

Variceal bleeding is the most severe complication of portal hypertension, with an estimated mortality of up to 30% [4]. In an attempt to compensate for the increase in overall pressure from portal hypertension, the body will shift blood flow away from the portal venous system to smaller, weaker veins in the body, most commonly those within the mucosa of the stomach and the esophagus, referred to as gastric and esophageal varices, respectively [2]. These veins within the stomach and the esophagus are not accustomed to the increased amount of blood volume and pressure that accompanies this increase in blood flow. Therefore, these veins will become swollen and are consequently at an increased risk of rupture and potentially life-threatening bleeding. The risk of bleeding is around 5% per year for small varices, and can be as high as 15% per year for large varices [2].

Somatostatin is an endogenous peptide hormone with various functions throughout the body, as it has inhibitory actions on growth hormone, glucagon, and insulin [6]. In GI bleeding secondary to portal hypertension, somatostatin works by reducing portal pressure and splanchnic blood flow [4,7]. Octreotide is a synthetic analog of somatostatin that shares similar pharmacologic activity, but has additional benefits of improved potency and duration of action compared to somatostatin. Octreotide is currently indicated in the United States for the treatment of acromegaly, carcinoid syndrome, and vasoactive intestinal peptide tumors, but it is also used off-label to help control gastrointestinal bleeding [4,5,6,7]. Octreotide is administered either intravenously, subcutaneously, or intramuscularly. Because octreotide is not yet approved for use in gastrointestinal bleeding secondary to portal hypertension, an effective dose for this indication has not been established. Octreotide is both absorbed and distributed rapidly, and it binds to lipoproteins and albumin, with an average of 41.2% of the drug bound in pharmacokinetic studies. Octreotide is then metabolized extensively by the liver and then eliminated primarily through the urine as approximately 32% unchanged drug. The use of octreotide in portal hypertension-induced bleeding has risen, as it has been found to have similar efficacy and lower adverse effects when compared to terlipressin. In addition to reducing splanchnic blood flow, terlipressin induces systemic vasoconstriction, which in turn decreases heart rate, cardiac output, and perfusion. This is associated with an increased risk of ischemia involving vital organs, including the heart, kidneys, and intestines, as well as peripheral ischemia. Octreotide has less systemic vasoconstriction and fewer hemodynamic effects, which suggests that it may be a preferable therapeutic alternative to terlipressin.

Most of the side effects of octreotide are due to the disruption in the growth hormone, glucagon, and insulin levels that follow its administration [6]. This disruption can cause abnormalities in blood glucose regulation, presenting as either hypoglycemia or hyperglycemia. These irregularities are often mild; however, there is a risk of developing severe hyperglycemia that could lead to the development of new-onset diabetes mellitus or an increased insulin requirement in those with pre-existing diabetes mellitus. Additionally, octreotide suppresses the secretion of thyroid-stimulating hormone, which can result in hypothyroidism. Other side effects include gallbladder abnormalities, bradycardia, arrhythmias, and GI side effects such as diarrhea, steatorrhea, vomiting, and abdominal bloating.

In the pediatric population, acute hemorrhage from portal hypertension is a medical emergency, as significant blood loss can lead to hemodynamic decompensation and shock. Currently, there are no universally accepted guidelines for the management of GI bleeding caused by portal hypertension in pediatrics, likely due to the limited amount of data in this population. The primary aim of this systematic review is to assess the utility of octreotide in treating portal hypertension-induced gastrointestinal bleeding in children. To the best of our knowledge, there are no formal systematic reviews on this topic have been published to date. Two narrative reviews addressing the use of octreotide in pediatric gastrointestinal disorders have been published: Mas et al. (2022) reviewed the evidence for octreotide use across multiple pediatric GI indications including GI bleeding, chylothorax, lymphangiectasia, pancreatitis, and dysmotility, concluding that GI bleeding represents the main indication based on adult and pediatric case series data [8], and Dunn et al. (2025) provided a comprehensive narrative review of practical applications of octreotide across GI and lymphatic disorders in children, highlighting the lack of prospective randomized trials and the need for standardized dosing guidance [9]. Neither review was conducted as a formal systematic review with a pre-registered protocol, structured PICOS framework, or risk of bias assessment, which distinguishes the present work.

This study highlights recent clinical findings examining the efficacy and safety of octreotide use in this critical condition. Through a detailed analysis of the available data, this review aims to provide evidence-based insights to guide healthcare professionals in making informed treatment decisions and improving patient outcomes.

From a drug development perspective, the use of octreotide in pediatric portal hypertension is a clear example of drug repurposing, whereby an established medication with a known safety profile is redirected to address a severe condition lacking approved therapies. In pediatrics, such repurposing strategies are often necessitated by ethical, regulatory, and logistical barriers that limit the conduct of large randomized trials. This systematic review therefore examines octreotide not only as a therapeutic option but also as a case study in pediatric drug repurposing.

## 2. Methods

### 2.1. Study Design and Search Strategy

This systematic review was conducted in accordance with the 2020 Preferred Reporting Items for Systematic Reviews and Meta-Analyses (PRISMA) guidelines [10]. The review protocol was developed using the PICOS framework (population, intervention, comparison, outcomes and study design).

The search strategy combined medical subject headings (MeSH) and Boolean operators. The last literature search across PubMed, Embase, Web of Science, Cochrane Library, and EBSCOhost was conducted on 12 November 2025. An additional search of the ClinicalTrials.gov registry was conducted on 20 March 2026 to identify registered but unpublished studies. For PubMed, the following search was used: (portal hypertension [MeSH Terms]) AND (gastrointestinal hemorrhage [MeSH Terms]) AND (((pediatric [MeSH Terms]) OR (children [MeSH Terms]) OR (adolescent [MeSH Terms]) OR (infant [MeSH Terms]) OR (child [MeSH Terms]))) AND (((sandostatin [MeSH Terms]) OR (octreotide [MeSH Terms]) OR (octreotide acetate [MeSH Terms]))). For EmBase, the following search was used: ‘portal hypertension’/exp AND ‘gastrointestinal hemorrhage’/exp AND (‘pediatric’/exp OR ‘child’/exp OR ‘infant’/exp OR ‘adolescent’/exp) AND ‘octreotide’/exp. For Web of Science, the following search was used: ALL = (portal hypertension) AND (ALL = (gastrointestinal hemorrhage) OR ALL = (gastrointestinal bleed)) AND (ALL = (pediatric) OR ALL = (child) OR ALL = (children) OR ALL = (infant) OR ALL = (adolescent)) AND (ALL = (octreotide)). For the Cochrane Library, the following search was used: (MeSH descriptor: [Portal Hypertension]) AND (MeSH descriptor: [Gastrointestinal Hemorrhage] explode all trees) AND (MeSH descriptor: [Pediatrics] explode all trees OR MeSH descriptor: [Child] explode all trees OR MeSH descriptor: [Adolescent] explode all trees OR MeSH descriptor: [Infant] explode all trees) AND MeSH descriptor: [Octreotide] explode all trees. Lastly, for EBSCOhost, the following search was used: (portal hypertension) AND (gastrointestinal bleeding or gi bleeding or gi bleeds) AND (pediatric or child or children or infant or adolescent) AND (octreotide or sandostatin). For ClinicalTrials.gov, the following search was used: “portal hypertension” [Condition or Disease] AND “octreotide” [Intervention or Treatment] AND (“pediatric” OR “children” OR “child” OR “infant” OR “adolescent”) [Other terms].

### 2.2. Inclusion and Exclusion Criteria

Studies were included if they met the following criteria:

- Population: patients 18 years of age or younger with portal hypertension;
- Intervention: receiving octreotide for the treatment of gastrointestinal bleeding;
- Outcomes: reported data regarding safety (incidence of adverse events) and efficacy (bleeding recurrence and packed red blood cell requirements) in the treatment of gastrointestinal bleeding;
- Study Design: randomized controlled trials and observational studies.

Only full-text articles written in English were included. The search was limited to studies published in English to ensure accurate interpretation of clinical data during screening and data extraction.

Systematic reviews, case reports, case series, conference papers, and other non-primary research were excluded from this review. Studies without available results or full text, duplicate articles, or those reporting clinically irrelevant outcomes were also excluded. Reference lists of included studies and relevant reviews were also screened to identify additional eligible publications.

Two independent reviewers screened studies in an unblinded manner. Titles and abstracts were assessed for relevance, followed by full-text screening based on the criteria above. Disagreements were resolved through consensus or consultation with a third reviewer.

Duplicates were removed semi-automatically using the free version of the Rayyan^®^ tool, followed by manual verification of authors and titles [11].

### 2.3. Inclusion of Studies Using Different Octreotide Formulations

The included studies employed different formulations and routes of administration of octreotide: two evaluated acute intravenous bolus or infusion therapy, while one investigated long-acting intramuscular octreotide for chronic management. Given the limited pediatric evidence on this topic, we chose to include all studies regardless of formulation. Though the clinical contexts and pharmacokinetics differ, the therapeutic goal—reducing gastrointestinal bleeding associated with portal hypertension—was consistent across studies. Excluding the long-acting formulation would have removed the only source providing pre- and post-treatment data, substantially limiting the interpretability of treatment effects. Therefore, all formulations were retained, with results synthesized qualitatively and heterogeneity explicitly acknowledged.

### 2.4. Data Collection

Two reviewers independently obtained clinical and demographic data from each included study. All data were cross-checked against one another, and discrepancies were resolved through discussion and further analysis. A third author oversaw the process to ensure consistency and accuracy.

Data collected included the age of participants, dose of octreotide received, sex of participants, bleeding recurrence, packed red blood cell requirements, and incidence of adverse effects.

### 2.5. Outcomes

The outcomes of interest were classified as follows:

- bleeding recurrence;
- packed red blood cell requirements;
- adverse events: any reported adverse events following octreotide treatment.

### 2.6. Synthesis Methods

All studies were grouped according to the reported outcomes, including bleeding recurrence, packed red blood cell requirements, and adverse events. Data were collected from each study and reported descriptively. Outcomes that were not reported consistently across all the studies were presented qualitatively. Data not reported in the included articles were treated as missing data. A systematic review was completed due to the heterogeneity of the limited data available.

### 2.7. Risk of Bias Assessment

The qualitative risk of bias of included non-randomized studies was assessed using the ROBINS-I (risk of bias in non-randomized studies—of interventions) version 2 tool [12]. ROBINS-I evaluates seven domains:

1. Confounding;
2. Classification of interventions;
3. Selection of participants;
4. Deviations from intended interventions;
5. Missing data;
6. Measurement of outcomes;
7. Selection of the reported result.

Two independent reviewers applied the ROBINS-I tool to each study, assigning a level of bias (low, moderate, serious, or critical) for each domain. The tool then provided guidance in assigning an overall risk of bias based on individual domain ratings. Any discrepancies between reviewers were resolved through discussion.

### 2.8. Certainty Assessment

Two reviewers independently assessed the certainty of the evidence using the GRADE (Grading of Recommendations Assessment, Development, and Evaluation) methodology. Each outcome was rated as very low, low, moderate, or high certainty based on study design, risk of bias, inconsistency, indirectness and imprecision.

GRADEpro software was used to generate a Summary of Findings (SoF) table [13]. Certainty ratings were downgraded when appropriate, and justifications were documented through accompanying comments.

## 3. Results

### 3.1. Study Selection

**Figure 1:**
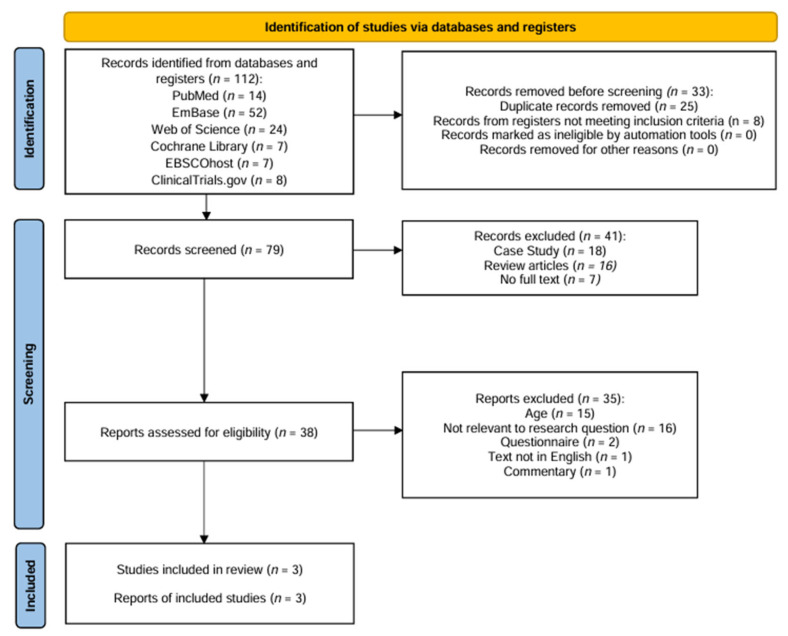
PRISMA flowchart of database search and article screening in the study [10].

Figure 1 displays the search strategy and study selection process through a PRISMA flow diagram [10]. A total of 112 records were identified through a comprehensive search of 6 sources, including 5 bibliographic databases (PubMed, Embase, Web of Science, Cochrane Library, and EBSCOhost; *n* = 104) and the ClinicalTrials.gov registry (*n* = 8). Of the 104 database records, 25 were identified as duplicates and were subsequently removed semi-automatically using the free version of the Rayyan^®^ platform, followed by manual verification of authors and titles. The 8 records from ClinicalTrials.gov did not meet the inclusion criteria and were excluded before screening. The remaining 79 articles underwent an initial screening of their titles and abstracts, where case studies, review articles, and those with no full text available were removed. The remaining 38 studies underwent full-text screening. A total of 35 additional articles were excluded based on inclusion of participants over the age of 18, results not being relevant to the research question, articles of questionnaire format with no relevant objective outcomes, articles not in English, and one commentary article. After screening, three articles were included in the final systematic review.

### 3.2. Baseline Characteristics of the Included Studies

Baseline characteristics of three included studies are summarized in Table 1 [14,15,16]. All studies were non-randomized cohort studies published from 2011 to 2015. The total number of patients included across all studies was 33, with a mean age of 6.3 years. The overall sex distribution was 57.6% male and 42.4% female. The routes of administration included intravenous and intramuscular, which varied between studies.

### 3.3. Concomitant Endoscopic and Supportive Therapies

Across the included studies, octreotide was frequently administered in conjunction with endoscopic and supportive treatments. In the study by Al-Hussaini et al., 4 patients (patients 4, 5, 6, and 8) underwent esophageal variceal sclerotherapy on the first day of octreotide treatment [15]. In the study by Meneses et al. [16], all patients underwent endoscopy during bleeding episodes, and endoscopic therapy was performed in 15 of 26 episodes (57.7%), including sclerotherapy and band ligation for esophageal varices, as well as cyanoacrylate injection for gastric varices. One episode involved concomitant sclerotherapy and cyanoacrylate treatment. Additionally, all patients received proton pump inhibitors or H2 receptor antagonists, and vitamin K replacement therapy was administered in 16 of 26 episodes (61.5%). In the O’Meara study [14], patients with identified esophageal and/or gastric varices underwent endoscopic interventions, including band ligation or sclerotherapy, following detection by upper gastrointestinal endoscopy.

### 3.4. Results of Individual Studies

The included studies reflect two distinct therapeutic strategies: acute management with short-acting intravenous octreotide and chronic management with long-acting intramuscular formulations. These approaches differ in both pharmacokinetics and clinical objectives and should therefore be interpreted separately when considering treatment effects.

#### 3.4.1. Rebleeding Occurrence

O’Meara et al. reported a statistically significant reduction in GI bleeding recurrence, with a median of 1.5 (range 0–27) GI bleeding events occurring after initiation of octreotide therapy compared to the median of 22 (range 12–110) GI bleeding events prior to octreotide initiation [14].

Al-Hussaini et al. noted that out of the 7 patients with portal hypertension, 3 patients had experienced bleeding recurrence (42.9%) [15].

Meneses et al. documented 3 out of 17 patients (17.6%) presented with rebleeding following octreotide infusion [16].

#### 3.4.2. Packed Red Blood Cell Requirements

O’Meara et al. recorded a significant decrease in the median number of blood units required before versus after octreotide administration as 5 units and 0.5 units, respectively [14].

Meneses et al. reported the median blood transfusion requirements following octreotide administration as 16.4 mL/kg [16].

#### 3.4.3. Adverse Events 

O’Meara et al. observed no serious adverse effects directly attributable to the long-acting octreotide treatment [14].

Al-Hussaini et al. documented that one patient developed hyperglycemia, with a blood glucose of 18 mmol/L, one week after initiating octreotide therapy [15]. The hyperglycemia was resolved following 10 days of insulin therapy.

Meneses et al. commented that hyperglycemia occurred in 5 out of 22 bleeding episodes treated with octreotide [16]. All cases of hyperglycemia were reversed quickly by lowering the IV octreotide dose rather than initiating insulin treatment (Table 2).

Interpretative note

The absence of adverse events in O’Meara et al. should be interpreted with caution given the retrospective and passive nature of data collection. Across all included studies, adverse event surveillance was not standardized or protocolized, potentially leading to underreporting and limiting the reliability of the safety conclusions.

### 3.5. Risk of Bias 

**Figure 2:**
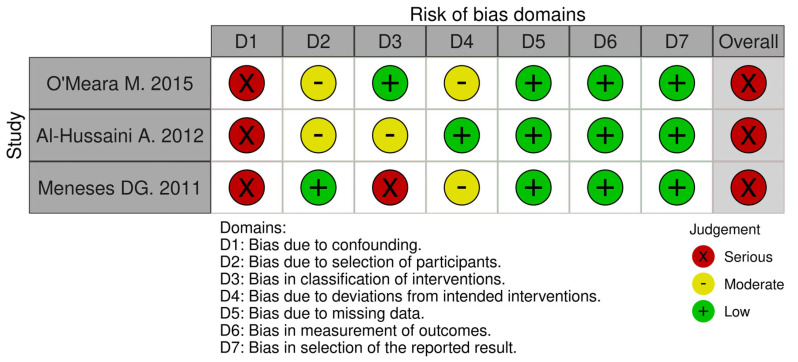
Risk of bias traffic light in non-randomized studies of interventions (ROBINS-I) assessments for the non-randomized studies included in this review. Each domain is judged as having low, moderate, serious or critical risk of bias [14,15,16].

**Figure 3:**
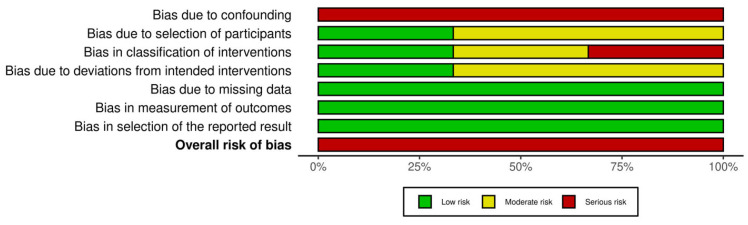
Aggregated overall risk of bias for all three sources.

Three non-controlled studies evaluating the effects of octreotide in pediatric patients with portal hypertension-induced gastrointestinal bleeding were included in the review (Figure 2). According to the ROBINS-I tool, all three studies included in this review exhibited a serious overall risk of bias [12]. This high overall risk (Figure 3) can be attributed to the lack of control for confounding variables in all of the studies. The studies did not control for comorbid conditions, concomitant medication use, or the severity of portal hypertension or GI bleeding in their patient populations. These confounding variables added a serious risk of bias to the reported results. This serious risk of bias is common in retrospective studies. Despite the lack of control of confounding variables, all three studies presented all planned outcome data and measured all results appropriately.

### 3.6. Certainty of Evidence

**Figure 4:**
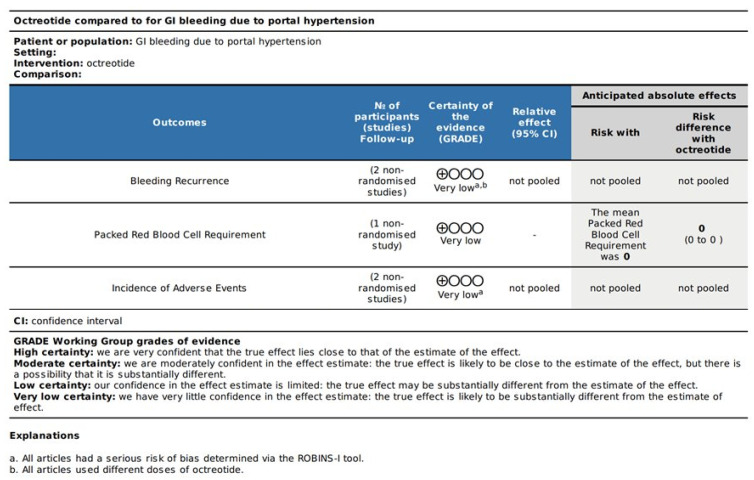
SoF of the certainty assessment with GRADE. The evidence is classified into high, moderate, low, and very low certainty.

GRADEpro software was used to create a Summary of Findings table (Figure 4). This table presents the overall certainty of the evidence reported for each of the three outcomes. All the evidence was determined to be of very low certainty. The evidence was downgraded due to the lack of control of confounding variables, small sample sizes, differences in doses of octreotide used, differences in the route of administration, and the differences in reported units of the outcomes.

## 4. Discussion

This systematic review evaluated the safety and efficacy of octreotide in gastrointestinal bleeding from portal hypertension in the pediatric population. Three non-randomized studies were analyzed for the following outcomes: bleeding recurrence, packed red blood cell requirement, and incidence of adverse effects.

The findings of this review should be interpreted within the broader context of drug repurposing in pediatrics. Octreotide, originally developed and approved for endocrine and neuroendocrine indications, has been adopted off-label in pediatric portal hypertension based largely on adult data and real-world clinical experience. This reflects a common paradigm in pediatric pharmacotherapy, where urgent clinical need often precedes formal regulatory approval.

Importantly, the available evidence reflects real-world clinical effectiveness rather than efficacy. All included studies are small observational cohorts conducted in routine clinical settings, without the controlled conditions typical of phase I–III clinical trials. As a result, the current literature does not allow for a formal assessment of efficacy, and the findings should be interpreted as descriptive of real-world use under heterogeneous and potentially confounded conditions.

Octreotide had positive effects regarding bleeding recurrence in the O’Meara et al. study, with a significant reduction in median GI bleeding events from 22 (range 12–110) to 1.5 (range 0–27) after octreotide was started [14]. Because the studies conducted by Al-Hussaini et al. and Meneses et al. did not include information on the incidence of bleeding recurrence in their participants prior to octreotide administration, it is difficult to assess if octreotide had improved the rates of bleeding recurrence in those individuals [15,16].

The packed red blood cell requirement was reported in two of the included articles. However, the blood requirements were reported in differing units following different observational periods. For example, the O’Meara et al. study analyzed blood requirements 12 months both before and 12 months after octreotide administration, and they reported packed red blood cell requirements in blood units [14]. Conversely, Meneses et al. published the blood requirements in ml/kg for only the 15 days following octreotide administration [16]. Due to the differences in observation periods and the units reported, the results from these studies cannot be directly compared and, therefore, must be interpreted individually.

O’Meara et al. indicated that octreotide administration decreased the packed red blood cell requirements for patients with GI bleeding from portal hypertension from 5 units before administration to 0.5 units after [14]. This significant reduction presented in this study makes it reasonable to associate octreotide with the decrease in packed red blood cell requirement following its administration.

Meneses et al. presented the packed red blood cell requirements as the median requirement of 16.4 mL/kg for all participants following administration [16]. This value alone does not have much clinical meaning since no comparator was provided; therefore, it is difficult to make interpretations from this value alone. No true association between octreotide administration and packed red blood cell requirements can be made from this study. Octreotide was generally well tolerated, with no serious adverse events reported in any of the studies [14,15,16]. The only adverse effect observed by the investigators was hyperglycemia. O’Meara et al. reported no adverse events attributable to OCT-LAR treatment [14]. Hyperglycemia was reported in 1 of 7 patients in the Al-Hussaini et al. study [15] and in 5 of 22 evaluable bleeding episodes in the Meneses et al. study [16]. This side effect was relatively uncommon and easily reversed by lowering the octreotide dose or providing short-term insulin. Adverse event reporting was passive and retrospective in two of the three studies [14,15]; only Meneses et al. implemented prospective active monitoring of glycemia, blood pressure, and symptoms [16].

### 4.1. Limitations

There are several limitations to this systematic review. To begin, though an extensive literature search was conducted, only three articles matched the determined inclusion criteria. Many of the articles initially screened included participants over the age of 18 or those with GI bleeding not associated with portal hypertension. In addition, a significant portion of the literature available was in individual case report format. Notably, there were only 33 participants across all three of the studies that fit the determined inclusion criteria. Out of all participants, only 26 had data reported for all three outcomes. This small total sample size limited the ability to make meaningful conclusions, as it is difficult to confidently make therapeutic decisions based on three small observational studies.

Case reports and case studies were excluded from this systematic review because they generally provide a low level of evidence for drawing reliable conclusions. These reports typically involve one or a very small number of participants, which limits the ability to generalize findings to a broader population. Additionally, case reports do not include control groups, making meaningful comparisons difficult and increasing the risk of bias. Therefore, this systematic review included only randomized controlled trials and observational studies with appropriate sample sizes and defined comparison groups to ensure a higher quality of evidence.

Also, the search was restricted to English-language publications, which may have introduced language bias and result in the exclusion of relevant studies published in other languages. Indeed, although reference lists of included studies and reviews were screened, a systematic search of gray literature sources, such as conference abstracts, was not performed. Therefore, potentially relevant unpublished or non-indexed data may not have been captured.

A key limitation of this review is the heterogeneity introduced by including studies that used different octreotide formulations in distinct treatment settings. The long-acting intramuscular formulation was administered for chronic control of recurrent bleeding, whereas the intravenous formulations were used for acute hemorrhage. These differences in pharmacological profile and therapeutic intent reduce the comparability of outcomes and limit the strength of any overall conclusions. Though most of the studies shared outcomes, they presented their data in differing metrics. These inconsistencies between units prevented statistical analysis from being conducted. While retaining all studies was necessary to preserve the limited available evidence, this heterogeneity restricts the ability to draw definitive statements regarding efficacy.

In addition, the frequent use of concomitant endoscopic and supportive therapies, including variceal banding, sclerotherapy, acid suppression, and vitamin K supplementation, is a major confounding factor. These interventions may have independently influenced bleeding control and transfusion requirements, limiting the ability to isolate the effect of octreotide. Other confounding variables, such as severity of disease, comorbid conditions, and use of other medications can also influence the results.

Lastly, there was missing data across all of the reports preventing a quantitative synthesis from being conducted. All primary authors were contacted by e-mail in an attempt to obtain the necessary data for analysis. Specifically, O’Meara [14] was asked to provide the number of patients who experienced bleeding recurrence and to report packed red blood cell requirements in units of mL/kg. In addition, Al-Hussaini [15] was contacted regarding the missing packed red blood cell requirement data for patients with portal hypertension. Meneses [16] reported numerical data but did not include a comparator for packed red blood cell requirements, so this information was also requested. Unfortunately, the requested data were not received. Furthermore, the available data were reported in differing units and, in some cases, lacked appropriate comparators, which made it difficult to perform a quantitative analysis.

### 4.2. Implications for Practice and Future Research

Current findings suggest that octreotide can be a possible treatment option for children with GI bleeding from portal hypertension. However, the quality and quantity of available evidence are not sufficient to draw definitive conclusions regarding the safety and efficacy of octreotide in this population.

Well-designed randomized controlled trials with longer follow-up periods are urgently needed to assess long-term outcomes. Future studies should include large cohorts of children receiving standardized doses of octreotide, with careful pre- and post-administration monitoring. Outcomes should be measured objectively, for example through hemoglobin levels and packed red blood cell requirements, to allow robust conclusions regarding safety and efficacy.

The pediatric population presents unique challenges that may justify a more proactive use of octreotide. In children, portal hypertension is often secondary to extrahepatic portal vein obstruction (EHPVO) rather than cirrhosis, resulting in a different hemodynamic profile and potentially a more robust response to splanchnic vasoconstriction. Emerging evidence suggests that the therapeutic window for octreotide may extend beyond acute management [17].

Transition from continuous intravenous infusions to long-acting formulations has been successful in pediatric patients with refractory or recurrent variceal bleeding, significantly reducing bleeding episodes and hospitalizations [14]. This “salvage therapy” approach indicates that octreotide may stabilize portal pressure in cases where daily oral medications, such as non-selective beta-blockers, are limited by compliance or side effects.

Future research should focus on standardized dosing for octreotide LAR and prospective trials comparing octreotide with established pharmacologic therapies, such as non-selective beta-blockers, to evaluate efficacy and safety in pediatric portal hypertension. Additionally, studies may explore its role as a bridge to surgical interventions (e.g., meso-Rex bypass or TIPS), optimizing growth and nutrition before definitive procedures. The development of long-acting octreotide formulations further illustrates how drug repurposing can extend beyond acute management, potentially reshaping treatment pathways and reducing healthcare burden without the need for de novo drug development.

## 5. Conclusions

Octreotide is a plausible therapeutic option for use in portal hypertension-induced gastrointestinal bleeding in children. Due to the limited number of relevant studies available and the observational nature of each, no strong conclusions can be drawn regarding the safety or efficacy of octreotide in this patient population. The results reported in this review should be interpreted with caution, as there was a serious risk of bias for all three articles, and very low certainty of evidence for each outcome. Further clinical trials in this population are necessary in order to guide medical practice.

## 6. Declarations

### Registration and Protocol

PROSPERO 2025 CRD420251245186. Available at https://www.crd.york.ac.uk/PROSPERO/view/CRD420251245186 (access on 4 December 2025).

## Figures and Tables

**Table 1:** Baseline characteristics of the included studies.

Study	Year	Design	Observation Period	Octreotide Dose (Median, Range)	No. of Patients	Age (Median, Range)	Sex [n (%)]
O’Meara et al. (J Pediatr Gastroenterol Nutr) [14]	2015	Single-center retrospective study	24 months (12 months before octreotide treatment and 12 months after octreotide treatment)	0.38 mg/kg (0.29–0.95 mg/kg) IM monthly	9	1.8 years (0.1–14.5 years)	M: 7 (77.8%) F: 2 (22.2%)
Al-Hussaini et al. (Saudi J Gastroenterol) [15]	2012	Single-center retrospective study	Up to 14 months	2 mcg/kg (1–5 mcg/kg) IV bolus; 2 mcg/kg/h (1–5 mcg/kg/h) IV infusion	7 *	3.0 years (0.5–13 years)	M: 4 (57.1%) F: 3 (42.9%)
Meneses et al. (Rev Paul Pediatr) [16]	2011	Single-center retrospective study	15 days following the end of octreotide infusion	1 mcg/kg (0.5–2.63 mcg/kg) IV bolus; 0.92 mcg/kg/h (0.44–1.43 mcg/k/h) IV infusion	17	7.9 years (0.6–18.9 years)	M: 8 (47.1%) F: 9 (52.9%)

* Including patients with portal hypertension.

**Table 2:** Adverse events reported across included studies.

Study	Formulation and Route	PHT Patients (n)	Adverse Event	Frequency	Severity	Timing	Management	Surveillance
O’Meara et al., 2015 [14]	OCT-LAR (IM, monthly)	9	None attributable to treatment	0/9 (0%)	—	—	—	Passive (retrospective clinical notes)
Al-Hussaini et al., 2012 * [15]	IV bolus + continuous infusion	7 *	Hyperglycemia	1/7 (14.3%)	Severe (18 mmol/L), requiring insulin	~1 week after initiation	Insulin (10 days), then discontinued	Passive (retrospective records)
Meneses et al., 2011 ** [16]	IV bolus + continuous infusion	17	Hyperglycemia	5/22 episodes (22.7%) **	Mild (max 169 mg/dL)	During infusion	Dose reduction; no insulin required	Active (prospective monitoring)

Notes. * Al-Hussaini et al. enrolled 21 patients for multiple gastrointestinal indications; only the 7 patients with portal hypertension-related bleeding were included in this review. Other adverse events reported in the full cohort (QT prolongation, growth hormone deficiency, hypertension) occurred exclusively in non-PHT patients and are not reported here. ** In Meneses et al., adverse events were reported per bleeding episode (n = 26); 4 episodes were excluded because octreotide was administered with a glucose solution, leaving 22 evaluable episodes.

## Data Availability

The original contributions presented in this study are included in the article Further inquiries can be directed to the corresponding authors.
